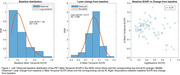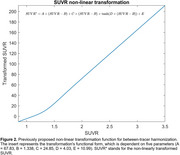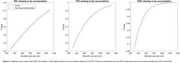# Impact of non‐linear SUVR transformations on the statistical power of tau‐PET longitudinal studies

**DOI:** 10.1002/alz70861_108621

**Published:** 2025-12-23

**Authors:** Alexis Moscoso Rial, Antoine Leuzy, Hartmuth C. Kolb, Victor L. Villemagne, Gregory Klein, Brian J Lopresti, Alexandra Gogola, Philip S. Insel, Diane Stephenson, Nadine Tatton, Yashmin Karten, Fang Xie, Christopher C. Rowe, Vincent Dore

**Affiliations:** ^1^ Critical Path Institute, Tucson, AZ USA; ^2^ Instituto de Investigación Sanitaria de Santiago de Compostela, Santiago de Compostela, Travesía da Choupana s/n Spain; ^3^ Enigma Biomedical Group, Knoxville, TN USA; ^4^ University of Pittsburgh School of Medicine, Pittsburgh, PA USA; ^5^ University of Melbourne, Melbourne, VIC Australia; ^6^ Austin Health, Melbourne, VIC Australia; ^7^ F. Hoffmann‐La Roche Ltd, Basel Switzerland; ^8^ University of Pittsburgh, School of Medicine, Pittsburgh, PA USA; ^9^ University of California, San Francisco, San Francisco, CA USA; ^10^ Huashan Hospital, Fudan University, Shanghai, Shanghai China; ^11^ Australian Dementia Network (ADNeT), Melbourne Australia; ^12^ CSIRO, Melbourne, VIC Australia

## Abstract

**Background:**

The growing availability of tau‐positron emission tomography (PET) radiotracers have facilitated the integration of tau‐PET into clinical trials. To enable reliable comparisons across tracers, several harmonization approaches, including the CenTauR scale, have been proposed. These methods differ mainly in how they model relationships between tracers, with the CenTauR scale applying a linear transformation while others using non‐linear functions. Although non‐linear transformations may better capture tracer relationships, they can alter statistical properties, potentially reducing statistical power. In this study, we evaluate the impact of applying a previously proposed non‐linear transformation for tau‐PET data in longitudinal tau‐PET studies assessing therapeutic effects on tau accumulation.

**Method:**

We included all amyloid‐β positive (>24 Centiloids) cognitively impaired individuals from the Alzheimer’s Disease Neuroimaging Initiative (ADNI) with available tau‐PET scans ([^18^F]flortaucipir) at baseline and at 1‐year follow‐up (n = 71; 41 with mild cognitive impairment and 30 with mild Alzheimer’s disease dementia). This cohort was used to estimate the parameters for power calculations, including 1‐year change‐from‐baseline metrics (Figure 1), in a population comparable to current clinical trial populations. Based on these parameters, we conducted simulations (N = 10,000 trials) to estimate the statistical power to detect a 50%, 75%, or 100% slowing of tau accumulation in a hypothetical therapeutic trial, comparing the use of raw SUVR values versus SUVR metrics transformed by a previously proposed non‐linear function (Figure 2).

**Results:**

Compared to raw SUVR, the non‐linear transformation consistently reduced statistical power (Figure 3), with a maximum difference of approximately 2%. Effect sizes were slightly lower with the non‐linear transformation (e.g., 50% slowing: d = 0.0996 for SUVR vs d = 0.0979; 100% slowing: d = 0.1995 for SUVR vs d = 0.1956). In 4.5% of the trial simulations, the transformation led to discordant conclusions regarding statistical significance (p < 0.05).

**Conclusion:**

Non‐linear transformations of tau‐PET SUVR data can modestly reduce the statistical power to detect treatment effects in clinical trials using tau‐PET as an endpoint. Future work will assess the impact of non‐linear transformations in other trial populations and study settings.